# Comparative study on landslide susceptibility mapping based on unbalanced sample ratio

**DOI:** 10.1038/s41598-023-33186-z

**Published:** 2023-04-10

**Authors:** Li Tang, Xianyu Yu, Weiwei Jiang, Jianguo Zhou

**Affiliations:** 1 School of Civil Engineering, Architecture and Environment, Hubei University of Technology, Wuhan, Hubei Province People’s Republic of China; 2 Innovation Demonstration Base of Ecological Environment Geotechnical and Ecological Restoration of Rivers and Lakes, Hubei University of Technology, Wuhan, Hubei Province People’s Republic of China

**Keywords:** Natural hazards, Environmental sciences

## Abstract

The Zigui–Badong section of the Three Gorges Reservoir area is used as the research area in this study to research the impact of unbalanced sample sets on Landslide Susceptibility Mapping (LSM) and determine the sample ratio interval with the best performance for different models. We employ 12 LSM factors, five training sample sets with different sample ratios (1:1, 1:2, 1:4, 1:8, and 1:16), and C5.0, Support Vector Machine (SVM), Logistic Regression (LR), and one-dimensional Convolution Neural Network (CNN) models are used to obtain landslide susceptibility index and landslide susceptibility zoning in the study area, respectively. The prediction performance of the model is evaluated by the receiver operating characteristic curve area under the curve value, five statistical methods, and specific category precision. The results show that the CNN, SVM, and LR models in the sample ratio of 1:2 achieve better performance than on the balanced sample set, which indicates the importance of the unbalanced sample set in training the LSM modeling. The C5.0 model is always in a state of overfitting in this study and needs to be further studied. The conclusions put forward in this study help improve the scientificity and reliability of LSM.

## Introduction

**Figure 1:**
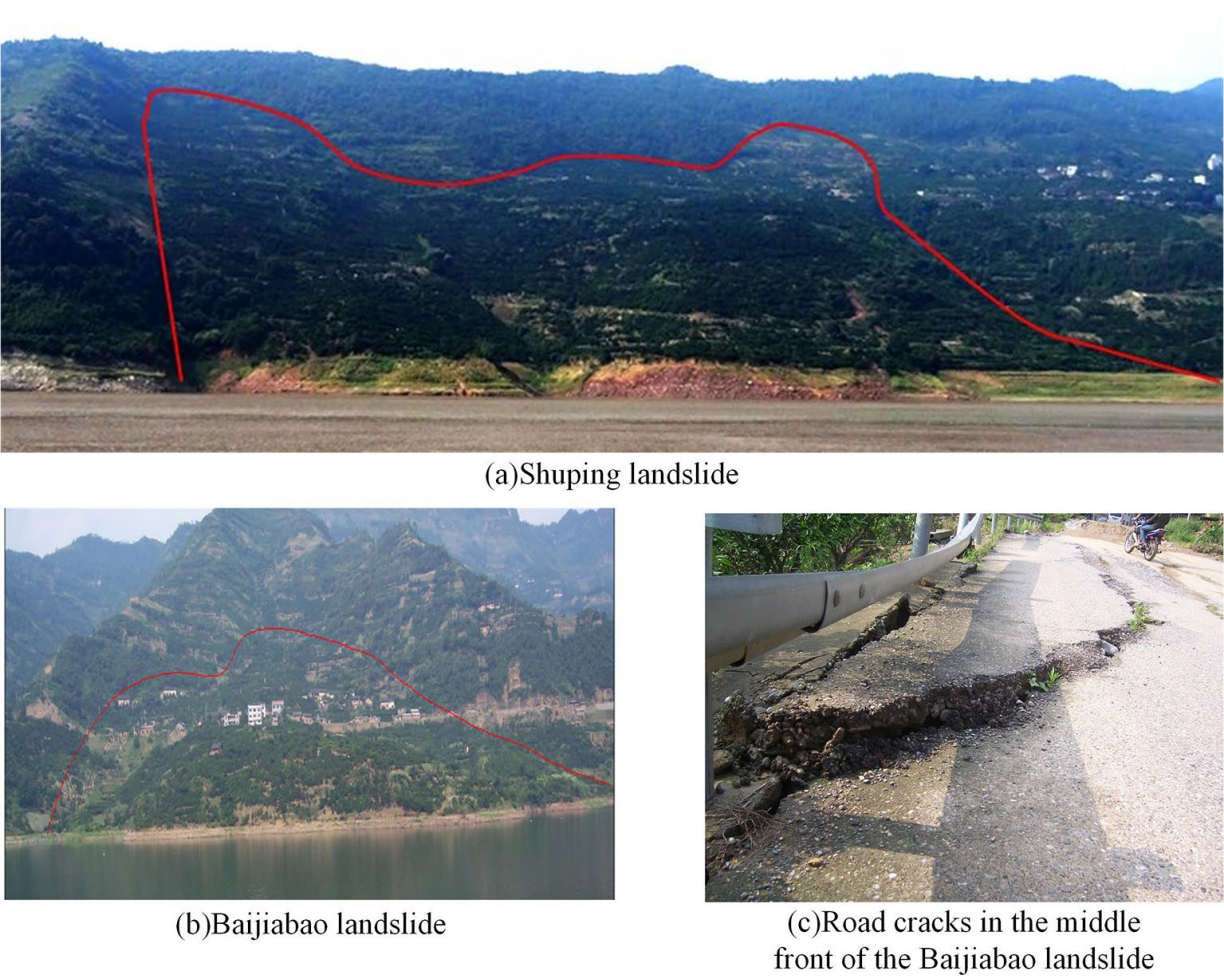
Example of landslide in the study area.

A large number of geological disasters occur worldwide every year, resulting in damage to human infrastructure and lives^1^. Landslides are geological disasters related to the movement of natural materials, usually accompanied by the movement of rocks and debris. Due to active geological movements, extreme changes in the global climate, and frequent human engineering activities, the landslide disasters in the Three Gorges Reservoir area (TGRA) have been increasing annually. There are also many new active landslides, which threaten the lives and property of residents on both sides of the TGRA, seriously affecting the shipping safety of the Yangtze River and reducing the service life of the reservoir^2,3^. The Shuping landslide and the Baijiabao landslide are typical of landslides in the study area, as shown in Fig. 1.

Landslide Susceptibility Mapping (LSM) is an effective tool for landslide disaster prevention and management, which can directly predict and describe the spatial distribution and probability of possible landslides^4^. With the rapid development of computer technology and Geographic Information Systems (GIS), an increasing amount of Machine Learning (ML) models have been introduced into LSM research, significantly enriching the application of quantitative methods in LSM^5–7^. Peng et al. successfully used rough set theory to extract the optimal LSM factor as the input of the SVM model, and the results showed that the prediction performance of the hybrid model was better than the general SVM model^8^. Mehrabi et al. combined genetic, particle swarm optimization, differential evolution, and ant colony optimization algorithms with an Adaptive Neuro-fuzzy Inference System (ANFIS), respectively, and used it for the spatial prediction of landslide distribution in Qazvin County in northwestern Iran. The accuracy of the ANFIS model after optimizing the calculation parameters with the above four types of algorithms can reach up to 91.6%^9^. Chen et al. compared the best first decision tree, random forest (RF), and naive Bayes tree models and evaluated the prediction ability of 14 factors, including elevation and slope. Finally, the Receiver Operating Characteristic (ROC) curve results showed that the RF model had the best performance, the maximum Area Under the Curve (AUC) value was 0.869, and the minimum standard error value was 0.025^10^. Although ML significantly improves accuracy and precision compared with traditional methods, it is not the optimal method for LSM due to issues of over-fitting, parameter adjustment, and low accuracy under the condition of sparse data^11^.

As an improved ML method, Deep Learning (DL) has been employed successfully in search technology, data mining, and other fields in recent years and has also made significant achievements in LSM^12^. Convolutional Neural Network (CNN) is a powerful DL technology designed by LeCun using the concept of updating parameters by gradient descent. CNN can autonomously learn the relationship between massive input and output data without needing to classify the input data, where latent rules are used to extract the local features of data for high-precision classification^13^. In 2019, Wang, Fang, and Hong first used CNN to extract features from factors for LSM in Yanshan County, China, constructing CNN-1D, CNN-2D, and CNN-3D for the spatial prediction of landslides. The three kinds of CNN convolutional networks were used and compared with the ML method of SVM. The experimental results showed that the CNN method greatly alleviated the problem of overfitting in ML and was more practical in LSM^14^. Yu et al. proposed an intelligent landslide detection algorithm based on deep CNN and an improved region-growing algorithm, where the experimental results confirmed the superiority of the algorithm in terms of detection accuracy and sensitivity^12^. Li, Fang, and Wang used a stacked ensemble to combine the CNN and Recurrent Neural Network (RNN) models. The hybrid framework was employed for landslide spatial prediction in the TGRA, obtaining a higher AUC value (0.918) than the single CNN model (0.904) and RNN model (0.900)^15^. Experiments have also demonstrated that the DL method has a superior prediction performance over traditional ML models, which is conducive to promoting the development of the theoretical and practical application of LSM^16,17^. However, these models adopt a 1:1 ratio of landslides and non-landslides when the training sample set is established; that is, the balanced sample (sample ratio is 1:1) set is used to train the model^18,19^, no discussion was developed for using unbalanced sample sets for LSM.

In the actual situation, the number of non-landslide samples is much higher than the number of landslide samples^20^. King and Zeng pointed out that the number of majority class events is usually two to five times more than minority class events in binary classification problems^21^. The ratio between the number of positive and negative samples in binary classification models was also found to affect the predictive performance of ML models^22^. Zhi, Guo, and Fan found that the prediction performance of the ML model depended on a large amount of training data^23^, and the sample size of the balanced sample set could not fully explain the diversity of LSM factors in the study area due to the small sample size so that the training model passively lost a lot of non-existent data. The important feature information of landslides made the prediction results of the trained model extremely dependent on random samples. While the prediction model trained using the balanced positive and negative sample data set had a good performance, the phenomenon that the same sample set had different AUC values made the LSM results unreliable. Wang et al. extracted 22 LSM factors and applied synthetic minority oversampling technology to the landslide dataset to solve the problem of unbalanced proportions of landslide and non-landslide sample sets. With the increase of samples, the performance of the four ML models of SVM, Logistic Regression (LR), artificial neural network, and RF all showed different degrees of improvement^24^. Zhang et al. used the class-weighted algorithm to transform the imbalance between landslide samples and non-landslide samples into a cost-sensitive problem. According to the results, the performance of the weighted model was better than that of the unweighted model and the class-weighted algorithm was suitable for solving the problem of unbalanced landslide samples in LSM^25^. The above scholars attempted to solve the problem of sample imbalance in LSM from the aspects of data processing or algorithm models and have achieved certain results^26,27^. However, they neglected to evaluate the proportion difference between the numbers of landslide samples and non-landslide samples in LSM, because for the traditional LSM, using the training sample training model with a same number of landslide samples and non-landslide samples is easy to cause false positives, resulting in the waste and loss of prevention costs, so it is necessary to further research on the impact of sample proportion on model prediction performance is required.

Based on the previous studies on LSM, this study employs LR, SVM, C5.0 decision tree (C5.0), and one-dimensional CNN models, taking the Zigui–Badong section of the TGRA as the study area. Five groups of landslide samples with a fixed number of landslide samples and a certain proportion of non-landslide samples are input into the training set (1:1, 1:2, 1:4, 1:8, and 1:16), providing five different LSM results. The impact of unbalanced sample sets on the LSM results is determined and discussed. One type of model can locate an optimal sample ratio interval and fully exploits the application potential of these four types of models in the actual LSM, providing a certain theoretical significance and scientific value for the research on LSM.

## Study area, data sources, and data processing platform

### Study area

**Figure 2:**
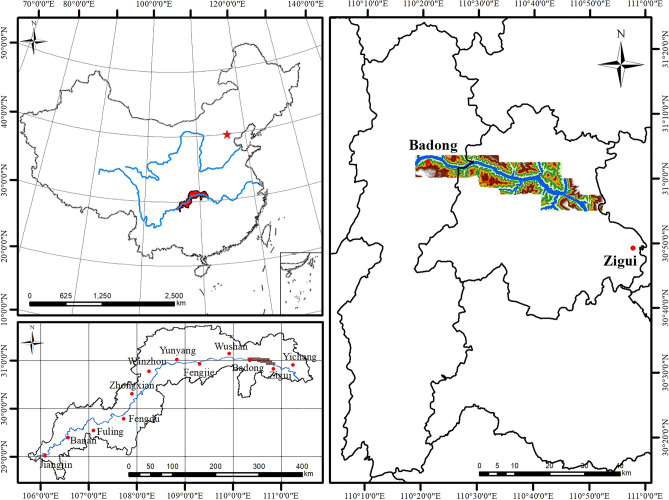
Geographical location of the study area (drawn with ArcGIS 10.8 software, and the URL is: https://www.esri.com/en-us/arcgis/about-arcgis/overview).

The study area is the Zigui to Badong section of the Three Gorges reservoir area, and the bank slope area extends 2–4 km along the Yangtze River to both sides, with an area of 388 km^2^. The longitude and latitude coordinates are 110° 18′ 44″–110° 52′ 30″ E and 30° 01′ 52″–30° 56′ 58″ N. The study area traverses two natural geographical units of the TGRA. The eastern part of the reservoir is the Three Gorges area in the Wushan Mountain range, and the western part is a low mountain and hilly area in eastern Pengdi of Sichuan Province. The terrain generally rises from southeast to northwest, with an elevation range of 80–2000 m. The geological structure features in the study area were formed between the late Yanshanian Movement and the early Himalayan Movement, and the main structural forms are folds and faults, including the Guandukou syncline in the west of Badong County and the Zigui syncline in the south of Xingshan County. The faults mainly include the Niukou, Xiangluping, Xiannushan, and Jiuwanxi faults from west to east. The strata in the study area are well-developed. The west of Xiangxi River is dominated by sandstone, shale, and other sedimentary clastic rocks, while the east of Xiangxi River is dominated by dolomite, limestone, and other carbonate rocks. The study area is in the mid-latitude zone, with a subtropical monsoon climate. The climate and rainfall change with the seasons and the temperature change affected by the elevation difference is obvious. The average annual rainfall in Badong County is 1034.3 mm, and the average annual rainfall in the Zigui area is 1158.9 mm. The location of the study area is shown in Fig. 2.

### Data and data processing platform

The data sources used in this study are shown in Table 1.

**Table 1:** Data sources were used in this study.

Name	Data source	Spatial resolution/Scale
DEM data	https://lpdaac.usgs.gov/tools/data-pool/	30 m
Basic geographic data	Hubei Geological Survey Institute^28^	1:50,000
Basic geological data	Hubei Geological Survey Institute^28^	1:50,000
Remote sensing data	https://earthexplorer.usgs.gov/	30 m
Atmospheric rainfall data	https://data.cma.cn	–
The landslides distribution data	Landslide hazard map^29^	1:10,000
China's border data	http://bzdt.ch.mnr.gov.cn/	–

Table 1 shows that the basic topographic and geological maps of the 1:50,000 scale and the landslide disaster map of 1:10,000 scale, DEM data and remote sensing data with resolution of 30 m. The average annual precipitation has a temporal resolution but does not have a spatial resolution^30^. All data layers were subsequently converted into the lowest resolution(30 m)^31^.

The following data processing platform used in this study:

- ArcGIS 10.8 (https://www.esri.com/en-us/arcgis/about-arcgis/overview);
- ENVI 5.3 (https://envi.geoscene.cn);
- SPSS Modeler 18 (https://www.ibm.com/products/spss-modeler);
- SPSS Statistics26 (https://www.ibm.com/products/spss-statistics);
- PyTorch 1.7.1 (https://pytorch.org).

### Landslide inventory mapping

The quantitative method for LSM is an engineering geological analogy method. Its core principle is to analyze and extract the spatial relationship between past landslides and LSM factors based on assuming that future landslides and existing landslides have the same environmental conditions and then to determine the distribution and probability of future landslides^32,33^. Through the investigation and study of Landsat 8 remote sensing image data, a landslide distribution map of 1:10,000 scale, a basic geological map of 1:50,000 scale, and a landslide survey report, a total of 202 landslides are identified in the study area. The landslides have a total area of 23.4 km^2^, accounting for 6.03% of the study area.

## Methods

### Factor analysis model

#### Pearson correlation coefficient analysis

The Pearson Correlation Coefficient (PCC) can be used to analyze the linear correlation between two variables. In the LSM, most of the factors are calculated by DEM and have natural correlation. Therefore, it is necessary to analyze and screen the factors through correlation analysis by PCC to ensure the mutual independence of the evaluation factors^34^. The calculation formula is shown in Eq. (1).

1 $$ \rho_{X,Y} = \frac{{cov\left( {X,{ }Y} \right)}}{{\sigma_{X} \sigma_{Y} }} = \frac{{E\left( {XY} \right) - E\left( X \right)E\left( Y \right)}}{{\sqrt {E\left( {X^{2} } \right) - E^{2} \left( X \right)} \sqrt {E\left( {Y^{2} } \right) - E^{2} \left( Y \right)} }} $$

where *cov* is the covariance, *σ*_*X*_ and *σ*_*Y*_ are the sample standard deviations, *E* is the mathematical expectation, and* X* and *Y* are a single sample point.

The value of the PCC is between − 1 and 1, which indicates that the correlation of these two variables is from negative correlation to positive correlation; when its value is 0, it means that the two variables have no correlation, that is, they are independent of each other. Two variables whose absolute value of correlation coefficient is greater than a certain threshold is usually regarded as two variables that are strongly correlated. When two variables have a strong correlation, one of them should be removed to eliminate the correlation^35^.

#### Multicollinearity analysis

It is necessary to perform a multilinear analysis before using the landslide factor dataset to train the model. The selection of evaluation factors directly affects the accuracy and reliability of LSM^36^. Multicollinearity analysis refers to judging whether an independent variable or multiple independent variables can be linearly combined into one independent variable, usually using Variance Inflation Factor (VIF) or Tolerance (TOL) to evaluate the evaluation factor multicollinearity. The formula for calculating the VIF value is shown in Eq. (2):

2 $$ {\text{VIF}} = \frac{1}{{\left( {1 - R_{j}^{2} } \right)}} $$

where *R*_*j*_^*2*^ is the coefficient of determination of the *j*-th independent variable to all other independent variables, and the TOL value is the reciprocal of the VIF value.

The larger the VIF value, the greater the possibility of collinearity between independent variables. Multiple covariance analysis is often used to evaluate the correlation between factors to ensure that there is no linear correlation between factors^37^. In LSM, if the VIF value of a factor is greater than 10 or the TOL value is less than 0.1, it means that the factor has serious multicollinearity problems, and the factor should be removed^38^.

### Relief-F analysis

Kira proposed a feature weighting Relief algorithm in 1992. The Relief-F algorithm evaluates the value of the LSM factor by calculating the correlation between the LSM factor and landslide, to determine the relative importance of the factor to the occurrence of landslide^36^. The principle is to assign different weights to features according to the correlation between each feature and category. When the weight of a feature is less than a certain threshold, the feature will be removed^39^. The Relief-F algorithm adds the ability to process multiple types of data on the basis of the original Relief algorithm, overcoming its limitation of only processing two types of data. The principle of Relief-F is to randomly select a sample *R* from the sample set *T*, find the *k* neighboring samples *H* of *R* from the sample set of the same class of *R*, and then find the *k* neighboring samples *N* of *R* from the sample set of different classes of each *R*, for all features, update the weights of features according to Eq. (3):

3 $$ W\left( A \right) = W\left( A \right) - \frac{{\mathop \sum \nolimits_{j = 1}^{k} diff\left( {A,R,H_{j} } \right)}}{{\left( {mk} \right)}} + \frac{{\mathop \sum \nolimits_{c \notin Class\left( R \right)} \left[ {\frac{p\left( C \right)}{{1 - p\left( {Class\left( R \right)} \right)}} \times \mathop \sum \nolimits_{j = 1}^{k} diff\left( {A,R,M_{j} \left( C \right)} \right)} \right]}}{{\left( {mk} \right)}} $$

where *diff(A, R*_*1*_*, R*_*2*_*)* represents the difference between the sample *R*_*1*_ and the sample *R*_*2*_ on the feature *A*, and the calculation formula is as follows:

4 $$ diff\left( {A,R_{1} ,R_{2} } \right) = \left\{ {\begin{array}{*{20}l} {\frac{{\left| {R_{1} \left[ A \right] - R_{2} \left[ A \right]} \right|}}{\max \left( A \right) - \min \left( A \right)},} \hfill & {if\;A\;is\;continuous} \hfill \\ {0,} \hfill & {if\;A\;is\;discrete\;and\;R_{1} \left[ A \right] = R_{2} \left[ A \right]} \hfill \\ {1,} \hfill & {if\;A\;is\;discrete\;and\;R_{1} \left[ A \right] = R_{2} \left[ A \right]} \hfill \\ \end{array} } \right. $$

When using the Relief-F method to evaluate the prediction ability of the landslide evaluation factor, the larger the value, the greater the weight of the evaluation factor feature, the stronger the influence ability of the feature, and the weaker the influence ability of the feature on the contrary^10^.

### Models

#### CNN model

**Figure 3:**
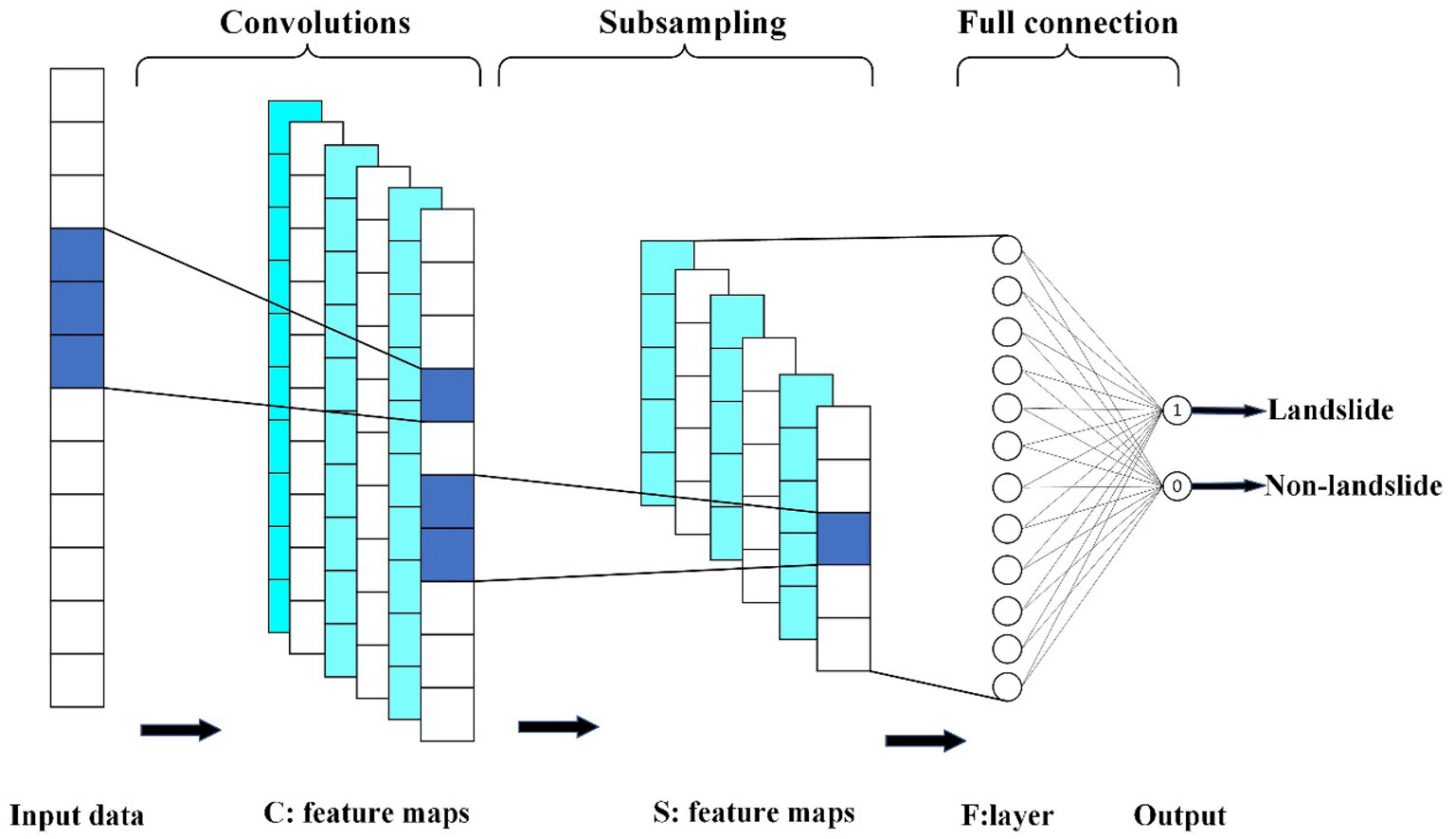
One-dimensional convolutional neural network structure, C represents the convolution layer, S represents the sampling layer, and F represents the fully connected layer.

The convolutional neural network used in this study employs the CNN-1D structure^14,40^, which consists of a convolutional layer, a maximum pooling layer, and a fully connected layer, as shown in Fig. 3. In the LSM, the neural network layer of the convolutional neural network can be used to directly learn the inherent laws and feature representations of landslide data. In this CNN-1D structure, the input data can be regarded as an image with only one column of pixels, and the number of pixels is determined by the number of landslide evaluation factors.

It is assumed that the kernel size of the convolutional layer of the one-dimensional CNN structure is m × 1, the kernel size of the maximum pooling layer is n × 1, a landslide evaluation factor is input, and after one convolution, the output length is (m-a + 1). The column vector S then enters the maximum pooling layer and outputs a column vector Y with a length of ((m − a + 1)/n). The column vector Y is connected to the fully connected layer with neural units to extract features. Finally, two neural units on the output layer give the result of the binary classification problem^14^.

#### C5.0 model

The C5.0 decision tree model selects attributes and sample partitions based on the information gain rate, and the information gain rate is derived from the concept of entropy. Some mathematical definitions in the C5.0 model are as follows:

5 $$ info\left( T \right) = - \mathop \sum \limits_{j = 1}^{k} \frac{{freq\left( {C_{j} ,T} \right)}}{\left| T \right|} \times log_{2}^{{\frac{{freq\left( {C_{j} ,T} \right)}}{\left| T \right|}}} $$

where *T* is a data set with n samples, the category attribute *C* contains *k* values (*C*_*1*_, *C*_*2*_, …, *Ck*), *freq*(*C*_*j*_, *T*) is the probability of occurrence of category *Cj*, and *T* is divided according to the attribute *X*. The conditional entropy of attribute *X* after segmentation is defined as follows:

6 $$ info_{X} \left( T \right) = \mathop \sum \limits_{i = 1}^{n} \frac{{\left| {T_{i} } \right|}}{\left| T \right|} \times info\left( {T_{i} } \right) $$

where |*T*_*i*_| is the number of examples in the data set *T* whose value is *c*_*i*_, and the information gain and information gain rate of the corresponding attribute *X* are:

7 $$ gain\left( X \right) = info\left( T \right) - info_{X} \left( T \right) $$

8 $$ gain\;ratio\left( X \right) = \frac{gain\left( X \right)}{{split\;info\left( X \right)}} $$

The calculation formula of *split info(X)* in the formula is shown in Eq. (9):

9 $$ split\;info\left( X \right) = - \mathop \sum \limits_{i = 1}^{n} \frac{{\left| {T_{i} } \right|}}{\left| T \right|} \times \log_{2} \left( {\frac{{\left| {T_{i} } \right|}}{\left| T \right|}} \right) $$

### SVM model

The support vector machine proposed by Vapnik is a supervised ML algorithm that constructs an n-dimensional hyperplane as a classification plane to classify the input data. Compared with other algorithms, SVM has the characteristics of using a smaller number of samples to obtain better classification results^41^. Assuming a non-linearly separable vector *x*_*i*_ (*i* = 1, 2, … , *n*), containing two types of *y*_*i*_ =  ± 1, the n-dimensional hyperplane is defined by Eq. (10):

10 $$ \left\{ {\begin{array}{*{20}c} {min\frac{1}{2}{\text{w}}^{2} } \\ {s.t.{\text{y}}_{{\text{i}}} \left( {\left( {{\text{w}} \times {\text{x}}_{{\text{i}}} } \right) + {\text{b}}} \right) \ge 1} \\ \end{array} } \right. $$

where *‖w‖* is the 2-norm of *w*, *w* is a vector perpendicular to the hyperplane, *x*_*i*_ is a point on the hyperplane, and *b* is a constant so that the hyperplane does not pass through the origin of the coordinate axes.

The training sample set is transformed into the n-dimensional space through the kernel function *K*(*x*_*i*_, *x*_*j*_), which is essentially a mapping function. The four commonly used kinds of kernel functions that satisfy the Mercer condition include linear, polynomial, radial basis, and sigmoid kernel functions. Chong studied the application of three sets of samples with different sizes combined with four types of kernel functions in LSM in earthquake areas and compared their prediction performance. Experiments demonstrated that the performance of the support vector machine model using the Radial Basis Function (RBF) kernel was better than other kernel SVM models^42^. Thus, we employ the SVM model based on the RBF kernel for LSM in this work.

### LR model

The LR model is a multivariate analysis model that effectively fits the relationship between dependent and independent variables. In this study, the dependent variable is the representation of whether there is a landslide (1 for landslide, 0 for non-landslide), and its expression is as follows^43^:

11 $$ z = \beta_{0} + \beta_{1} X_{1} + \beta_{2} X_{2} + \cdots + \beta_{n} X_{n} $$

where *z* is the dependent variable, $\left\{ {\beta_{0} ,\beta_{1} , \ldots ,\beta_{n} } \right\}$ is the regression variable, and $\left\{ {X_{1} ,X_{2} \ldots ,X_{n} } \right\}$ is the explanatory variable, then the calculation formula of the probability of occurrence *p* is as follows:

12 $$ p = \frac{1}{{1 + e^{ - z} }} $$

where *p* is a sigmoid function, and its value range is from 0 to 1. In this study, this value describes the probability of landslide occurrence.

### Model inputs and outputs

This study focuses on the influence of sampling methods on the results of LSM, so the inputs and outputs of the four models are inconsistent. The LSM factors and evaluation indicators are input to the model, the model is trained to obtain the relationship between the factors and the evaluation indicators, and then the validation sample is input, and the output is the landslide susceptibility index for each LSM calculation unit in the validation sample.

**Figure 4:**
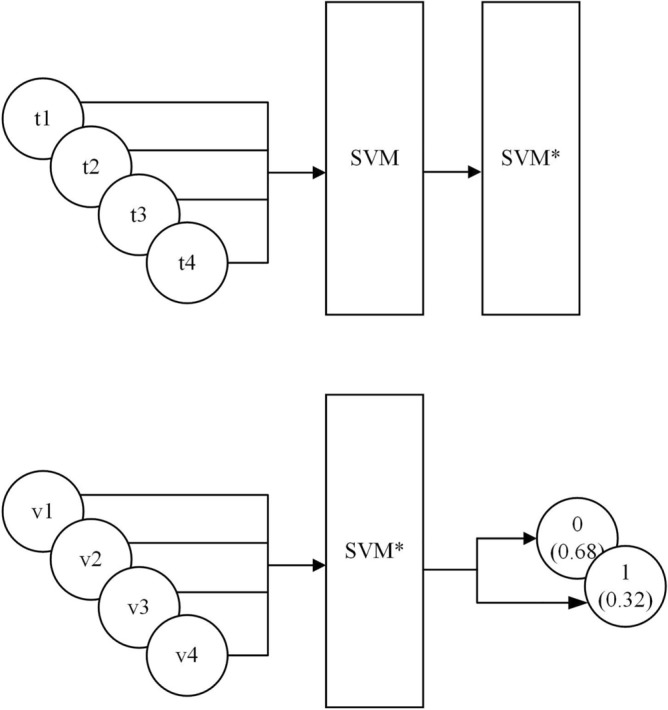
Schematic of the inputs and outputs of the model.

Taking the SVM model as an example, the input and output of the model are shown in Fig. 4.

Assuming that t1, t2, t3, and t4 are the four factors from the training sample set, the trained model SVM* is obtained by inputting them into the SVM model, after which v1, v2, v3, and v4 from the validation sample set are input into the SVM* model and the outputs are the probability distributions of 0 (non-landslide occurs) and 1 (landslide occurs), which are 0.68 and 0.32 respectively.

### Evaluation methods

#### ROC curve and AUC value analysis

The receiver operating characteristic curve is a common indicator used to verify the performance of the model, which can intuitively show the accuracy and reliability of the model prediction results. The ROC curve takes the true positive rate TPR as the Y-axis and the positive rate FPR as the X-axis, as defined in Eqs. (13) – (14):

13 $$ TPR = \frac{TP}{{\left( {TP + FN} \right)}} $$

14 $$ FPR = \frac{FP}{{\left( {FP + TN} \right)}} $$

where TP, FP, TN, and FN are defined by the confusion matrix. The verification of classification model performance plays a very important role in evaluating the generalization ability of LSM^44^. In the field of ML, the four types of comparison results between the predicted value and the actual value can be used as four types of indicators, as shown in Table 2.

**Table 2:** Confusion matrix.

Confusion matrix		Predict	
		Positive	Negative
True	Positive	True Positive, TP	False Negative, FN
	Negative	False Negative, FP	True Negative, TN

The four situations shown in the table are as follows: When the result is a landslide and the prediction is also landslide, it is a True Positive (TP); When the result is a non-landslide and the prediction is also non-landslide, it is a True Negative (TN); When the result is a non-landslide and the prediction is a landslide, it is a False Positive (FP); When the result is landslide and the prediction is non-landslide, it is a False Negative (FN).

For example, each point on the previous curve corresponds to a set threshold, and each different threshold corresponds to a different pair of TPR and FPR values. The closer the ROC curve is to the upper left corner, the better the classification effect of the classifier. In order to evaluate the performance of different LSM models under different conditions, the area under the curve is generally used as the evaluation standard^15,45^.

#### Five statistical methods

Overall accuracy (OA), precision, recall, F-measure, and Matthews correlation coefficient (MCC) are common indicators used to measure the ability of LSM classification models and are calculated from the confusion matrix^10,44^. The formulas of these five methods are denoted as Eqs. (15)–(19):

15 $$ OA = \frac{TP + TN}{{TP + FP + TN + FN}} $$

16 $$ Precision = \frac{TP}{{TP + FP}} $$

17 $$ Recall = \frac{TP}{{TP + FN}} $$

18 $$ F - Measure = \frac{2 \times Precesion \times Recall}{{Precision + Recall}} $$

19 $$ MCC = \frac{TP \times TN - FP \times FN}{{\sqrt {\left( {TP + FP} \right)\left( {TP + FN} \right)\left( {TN + FP} \right)\left( {TN + FN} \right)} }} $$

where TP, FP, TN, and FN are defined in the "Confusion Matrix" section.

#### Specific category precision analysis (SCPA)

In addition to the above-mentioned accuracy evaluation methods, this study also employs an improved method to evaluate the accuracy of various types of landslide susceptibility, which is called SCPA^30^. The traditional quantitative analysis method is based on the Landslide Susceptibility Zoning (LSZ), using landslide distribution data to calculate the proportion of landslide area in different types of LSZ, the analysis result is based on the proportion of the landslide area in very high susceptibility to the total area of the landslides. However, there is a problem with many areas in the LSZ belonging to the very high susceptibility LSZ, the model evaluation results are naturally good. Obviously, this cannot verify the effect of LSM. SCPA overcomes the above problem.

In this study, the SCPA method takes into account the number of calculation units in the classification area This method is defined as Eq. (20):

20 $$ p_{i} = \frac{{A_{i} }}{{B_{i} }} \times 100{\text{\% }} $$

where *i* = 1,2,…,*n*, *n* is the classification number of landslide-prone zonings, *A*_*i*_ is the number of slope units occupied by landslides in *i*-th LSZ classification, and *B*_*i*_ is the number of the slope units in *i*-th LSZ classification.

### Experimental process

**Figure 5:**
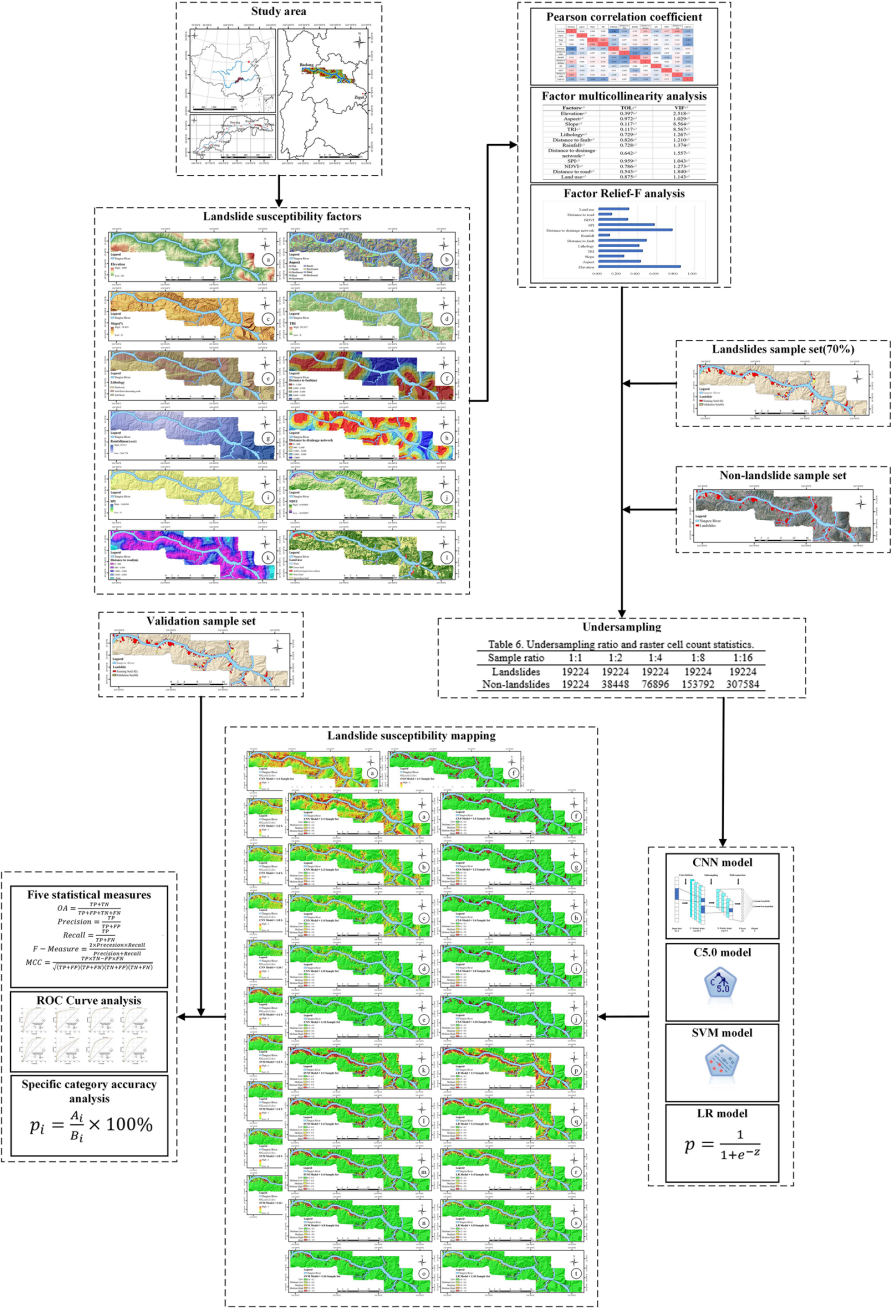
Flowchart of this study (drawn with ArcGIS 10.8 software, and the URL is: https://www.esri.com/en-us/arcgis/about-arcgis/overview).

The flowchart of this study is shown in Fig. 5.

The experimental process consists of three main steps. In the first step, 12 factors were selected and their correlations and relative importance were analyzed. The second step randomly selects training (70% of the total) and validation (30%) sets out of grid cells corresponding to landslide and non-landslide locations in the study area. Before establishing the LSM model, the undersampling method is used to process the training sample set. Five training sample sets were designed (the ratio of landslide samples to non-landslide samples were 1:1, 1:2, 1:4, 1:8, and 1:16). The final step constructs the LSM models to obtain different landslide susceptibility index (LSI) maps and LSZ maps. The ROC curve, five statistical evaluation methods, and SCPA are used for quantitative evaluations.

In addition, the quantization process of the 12 LSM factors is as follows:

- Calculate aspect, slope, TRI^46^, and SPI^9^ using spatial analysis tools in ArcGIS 10.8 software based on the digital elevation model;
- According to the topographic map and geological map, the lithology of the study area is divided into: hard rock, soft and hard alternation rock, and soft rock^36^;
- The distance to fault^47^, the distance to drainage network^48^, and the distance to road^49^ were obtained using the Euclidean distance method in the Spatial Distance Analysis Tool;
- The inverse distance weighting method was used to spatially interpolate the annual average rainfall data of each meteorological station to obtain the average annual rainfall in the study area^22^;
- Surface cover factors have a great influence on slope stability^50^. NDVI was calculated by using the red band and near-infrared band of a 30 m resolution image of LandSAT-8^40^. Meanwhile, remote sensing image was supervised and classified, and land use in the study area was divided into five categories: water, forest land, artificial impervious surface, grassland, and agricultural land.

## Result

**Figure 6:**
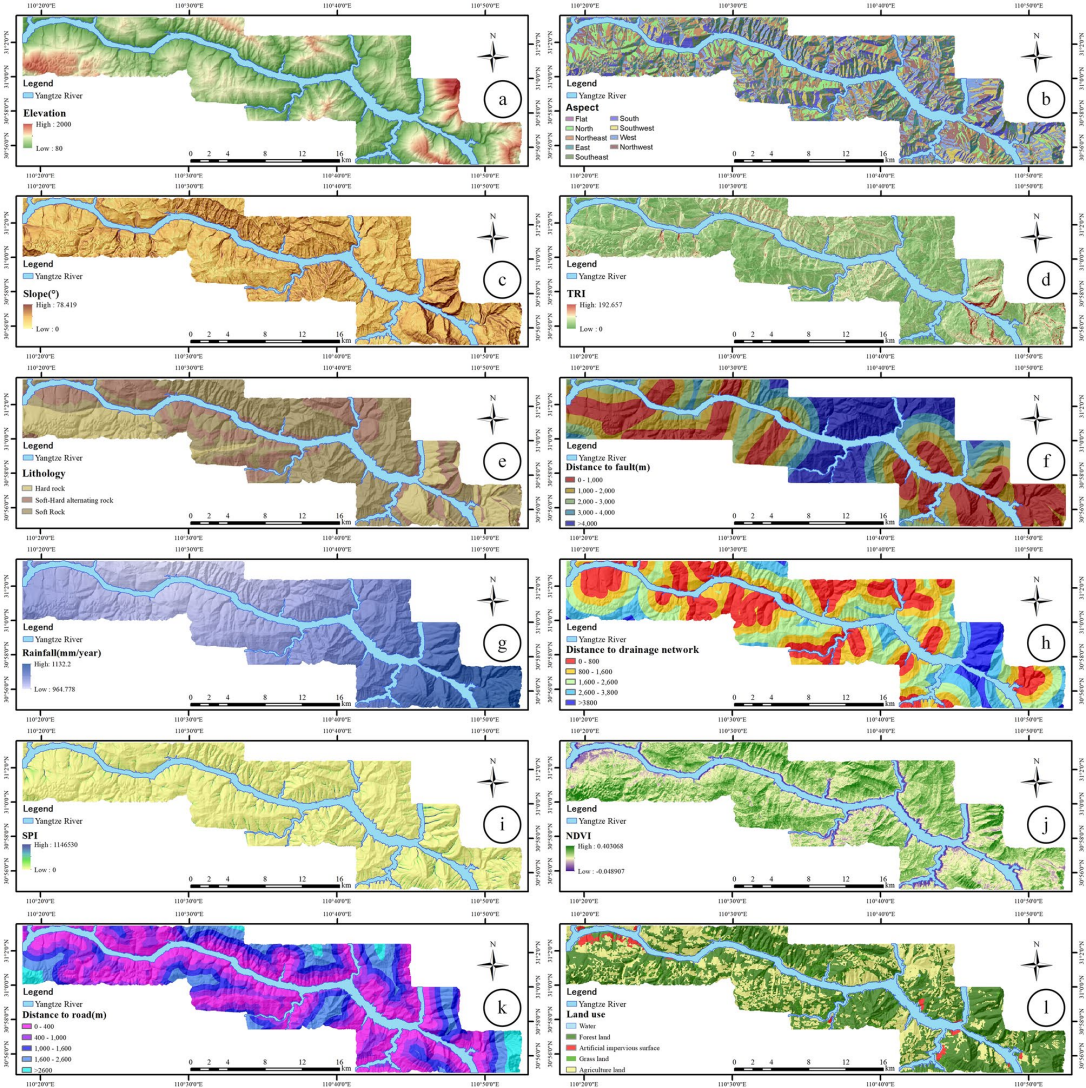
LSM factors in the study area (**a**) Elevation factor, (**b**) Aspect factor, (**c**) Slope factor, (**d**) TRI factor, (**e**) Lithology factor, (**f**) Distance to fault factor, (**g**) Rainfall factor, (**h**) Distance to drainage network factor, (**i**) SPI factor, (**j**) NDVI factor, (**k**) Distance to road factor, (**l**) Land use factor (drawn with ArcGIS 10.8 software, and the URL is: https://www.esri.com/en-us/arcgis/about-arcgis/overview).

The selection of appropriate LSM factors has a significant impact on the accuracy of LSM. Based on previous studies on the LSM in the TGRA from Zigui to Badong^30^, 12 LSM factors were selected and divided into topographic and geomorphological factors. These included elevation, slope, aspect, and terrain relief index (TRI)), geological factors (lithology, distance to faults), hydrological factors (average annual precipitation, distance to water system, stream intensity index (SPI)), and surface cover factors (normalized difference vegetation index (NDVI), distance to road, and land use type). The LSM factors are shown in Table 3 and Fig. 6.

**Table 3:** LSM factor selected in this study.

Category	Factor	Unit	Range	Type	Describe
Geomorphological factor	Elevation	m	80–2000	Continuous	Elevation represents the spatial variation of elevation, which can affect the degree of weathering of rocks and is an important factor in LSM^46^
	Aspect	–	(1) Flat, (2) North, (3) Northeast, (4) East, (5) Southeast, (6) South, (7) Southwest, (8) West, (9) Northwest	Discrete	The aspect is affected by solar radiation, weathering degree, and water evaporation, which affects the groundwater concentration and the stability of the slope^51,52^
	Slope	°	0–78.419	Continuous	The slope controls the balance between the retaining force and the unstable force acting on the slope. The steeper the slope, the more prone to landslides^22^
	TRI	–	0–192.657	Continuous	TRI defines the roughness of the topography of the study area, which affects topographic and hydrological processes that are critical to landslide development. It affects the incidence of landslides^46^
Geological factor	Lithology	–	(1) Hard rock, (2) Soft-hard alternation rock, (3) Soft rock	Discrete	Lithology is closely related to the spatial distribution of landslides. The softer the lithology, the higher the degree of weathering, and the easier it is to cause landslides^36^
	Distance to fault	m	0–8753.58	Continuous	The distance to the fault is an important LSM factor^53^, which has a negative impact on slope stability. Generally speaking, the farther the distance is, the less the number of landslides will occur. The distance to the fault plays a very important role in the formation of the landslide
Hydrological factor	Rainfall	mm/year	964.778–1132.2	Continuous	Rainfall is the most common triggering factor affecting landslides. Rainfall can penetrate along the cracks of the landslide body and seriously affect the shear strength of the slope. Generally, the heavier the rain, the more prone to landslides^54^
	Distance to drainage network	m	0–6078.24	Continuous	The distance to the drainage network is a key factor in the occurrence of landslides., it is composed of rivers and streams, which has a negative impact on the slope base and the underwater part of the slope^48^
	SPI	–	0–1,146,530	Continuous	SPI is a common hydrological factor in LSM studies, it describes the motion of strong grains of sediment by gravity and is an important topographic feature^55^
Surface cover factor	NDVI	–	0.048907–0.403068	Continuous	NDVI represents the growth of green vegetation in the study area, and vegetation coverage will have an important impact on the stability of the slope^56^
	Distance to road	m	0–4488.79	Continuous	Similar to the effect of distance to the drainage network, due to human activities, additional loads on the slope excavation cause slope changes, thereby affecting the slope stress state and balance^57^
	Land use	–	(1) Water, (2) Forest land, (3) Artificial impervious surface, (4) Grassland, (5) Agricultural land	Discrete	Land use factors have a great influence on slope stability^50^, which can also affect infiltration and runoff

**Figure 7:**
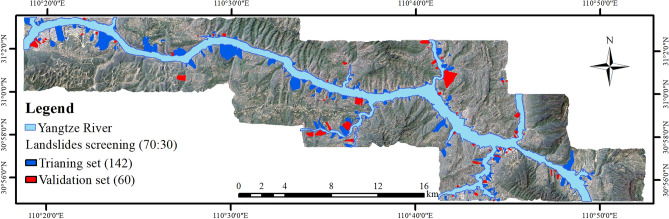
The division results of the training sample set and the validation sample set in this study (drawn with ArcGIS 10.8 software, and the URL is: https://www.esri.com/en-us/arcgis/about-arcgis/overview).

A 30 m × 30 m grid unit was used as the LSM unit, and the training sample and validation sample sets of the evaluation model were set. The grid cells of 202 landslides in the study area were screened out and marked as “1” and the grid cells in the non-landslide area were marked as “0”, with 25,606 grid cells and 398,977 grid cells, respectively. Considering each landslide as a whole, 70% of the landslides were randomly selected in the study area, providing 142 landslides (19,224 grid cells) as training samples, and the remaining 30%, or 60 landslides (6382 grid cells) as a verification sample. The division results are shown in Fig. 7. The landslides were selected as training samples, and all non-landslides formed the initial sample set, with a total of 418,201 grid cells.

**Figure 8:**
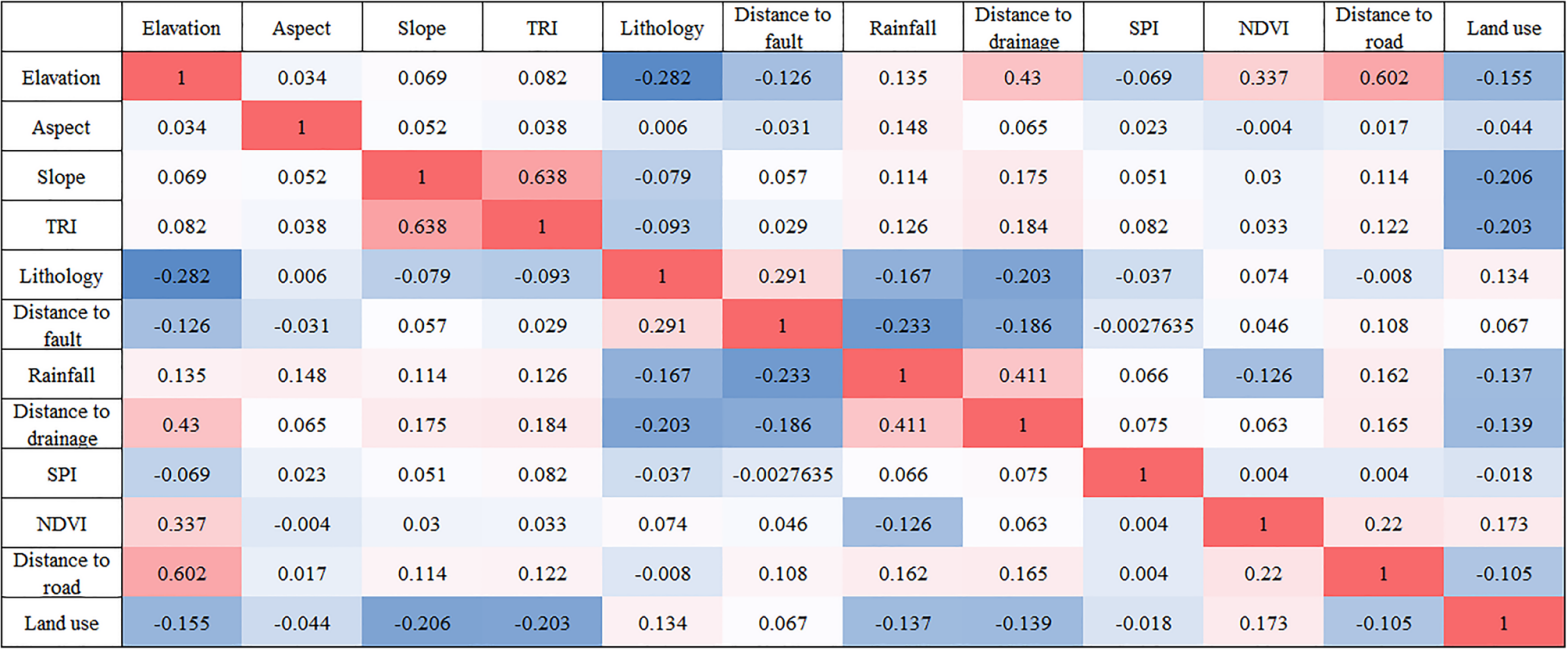
PCC matrix of 12 LSM factors.

Figure 8 shows the PCC calculation results of the 12 LSM factors, in this figure, the depth of color has different meanings, with darker red indicating a stronger positive correlation and darker blue indicating a stronger negative correlation.

Figure 8 shows that the correlation coefficients between the factors are all below. The result of slope and TRI is the highest, at 0.638, followed by elevation and distance to road, which is 0.602. Thus, all evaluation factors pass the PCC test.

The 12 LSM factors selected above were further analyzed using the variance expansion factor index, and the results are shown in Table 4.

**Table 4:** Multicollinearity of 12 LSM factors.

Factors	TOL	VIF
Elevation	0.397	2.518
Aspect	0.972	1.029
Slope	0.117	8.564
TRI	0.117	8.567
Lithology	0.729	1.267
Distance to fault	0.826	1.210
Rainfall	0.728	1.374
Distance to drainage network	0.642	1.557
SPI	0.959	1.043
NDVI	0.786	1.273
Distance to road	0.543	1.840
Land use	0.875	1.143

The largest TRI index in Table 4 has a VIF value of 8.567, all factors satisfy the condition of VIF < 10, and the 12 factors selected in this study pass the multicollinearity test^58^.

**Figure 9:**
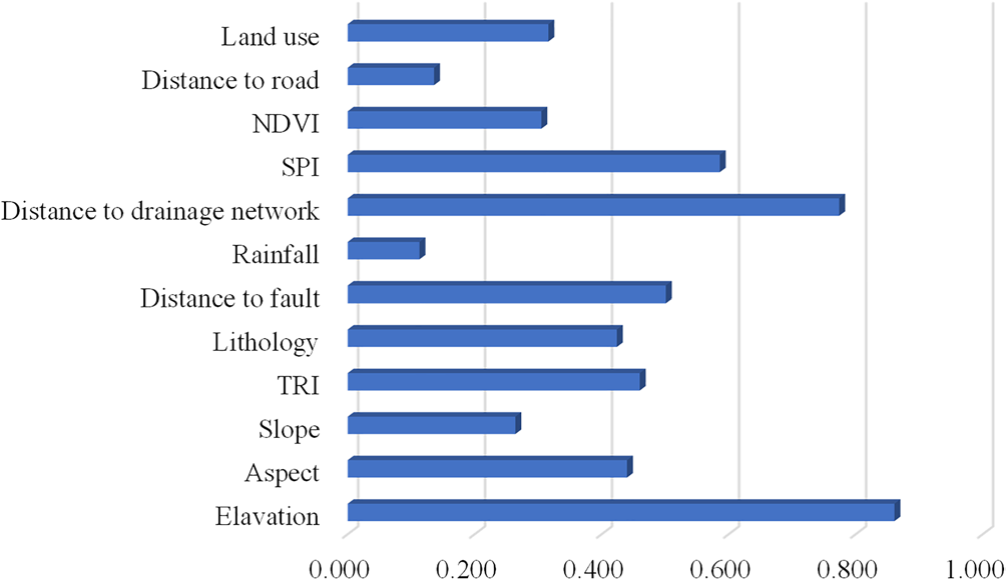
Relief-F coefficients of 12 LSM factors.

The calculation results using the Relief-F algorithm are shown in Fig. 9.

As illustrated, the Relief-F value of the average annual rainfall is the smallest (0.113), and the coefficient of this factor satisfies the condition of being greater than 0^40^. This result indicates that all the selected factors make important contributions in predicting landslides, so none are deleted.

### Sample set generation

Before establishing the LSM model, the undersampling method is used to process the non-landslide sample set. Thus, some data are deleted from the non-landslide sample set by a random non-manual intervention method, and the data volume of the non-landslide sample is reduced. In this study, the ratio of landslides selected as training samples to all non-landslides was 1:20.75. Five training sample sets were designed, which were a balanced sample set (the ratio of landslide samples to non-landslide samples was 1:1), and four unbalanced sample sets (the ratio of landslide samples to non-landslide samples were 1:1, 1:2, 1:4, 1:8, and 1:16). To conveniently represent the sample sets corresponding to different sample ratios, the former value in the sample set mentioned in this article represents a landslide, and the latter value represents a non-landslide. The proportion and number of these sample sets are shown in Table 5.

**Table 5:** Undersampling ratio and raster cell count statistics.

Sample ratio	1:1	1:2	1:4	1:8	1:16
Landslides	19,224	19,224	19,224	19,224	19,224
Non-landslides	19,224	38,448	76,896	153,792	307,584

### Experimental results of LSI

The parameter settings of the CNN-1D model used in this study were optimized by trial and error, in this CNN structure, *m* = 12, *a* = 3 *n* = 2. The optimized CNN-1D model parameters are shown in Table 6.

**Table 6:** Parameter setting of CNN model.

CNN parameters	Parameter setting
Kernel size	1 × 3
Max pooling layer kernel size	1 × 2
Activation function	ReLU
Optimizer	Adam
Learning rate	0.01
Batch size	2000

**Figure 10:**
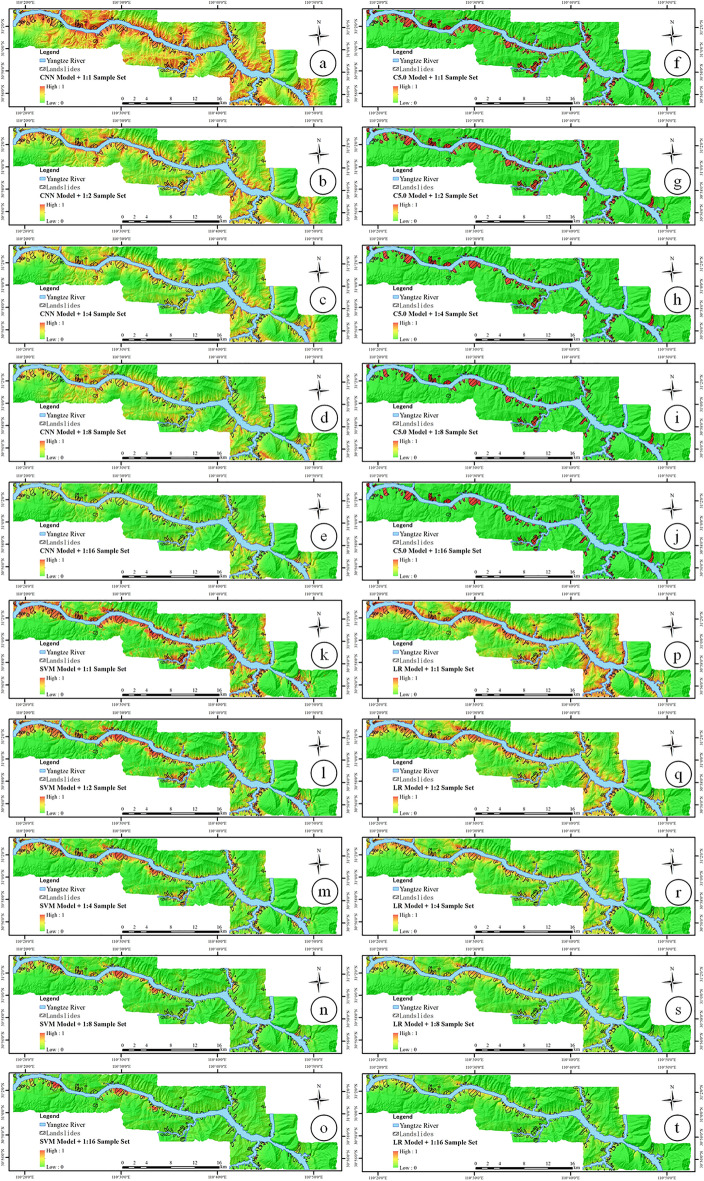
LSI based on (**a**) 1:1 sample set by CNN model, (**b**) 1:2 sample set by CNN model, (**c**) 1:4 sample set by CNN model, (**d**) 1:8 sample set by CNN model, (**e**) 1:16 sample set by CNN model, (**f**) 1:1 sample set by C5.0 model, (**g**) 1:2 sample set by C5.0 model, (**h**) 1:4 sample set by C5.0 model, (**i**) 1:8 sample set by C5.0 model, (**j**) 1:16 sample set by C5.0 model, (**k**) 1:1 sample set by SVM model, (**l**) 1:2 sample set by SVM model, (**m**) 1:4 sample set by SVM model, (**n**)1:8 sample set by SVM model, (**o**) 1:16 sample set by SVM model, (**p**) 1:1 sample set by LR model, (**q**) 1:2 sample set by LR model, (**r**) 1:4 sample set by LR model, (**s**) 1:8 sample set by LR model, (**t**) 1:16 sample set by LR model (drawn with ArcGIS 10.8 software, and the URL is: https://www.esri.com/en-us/arcgis/about-arcgis/overview).

The five groups of sample sets constructed above were respectively input into four types of models, and an LSM model was established to obtain the LSI in the study area. The LSI is a continuous value from 0 to 1. The experimental results are shown in Fig. 10.

### Evaluation of LSM results

#### ROC curve and AUC value

**Figure 11:**
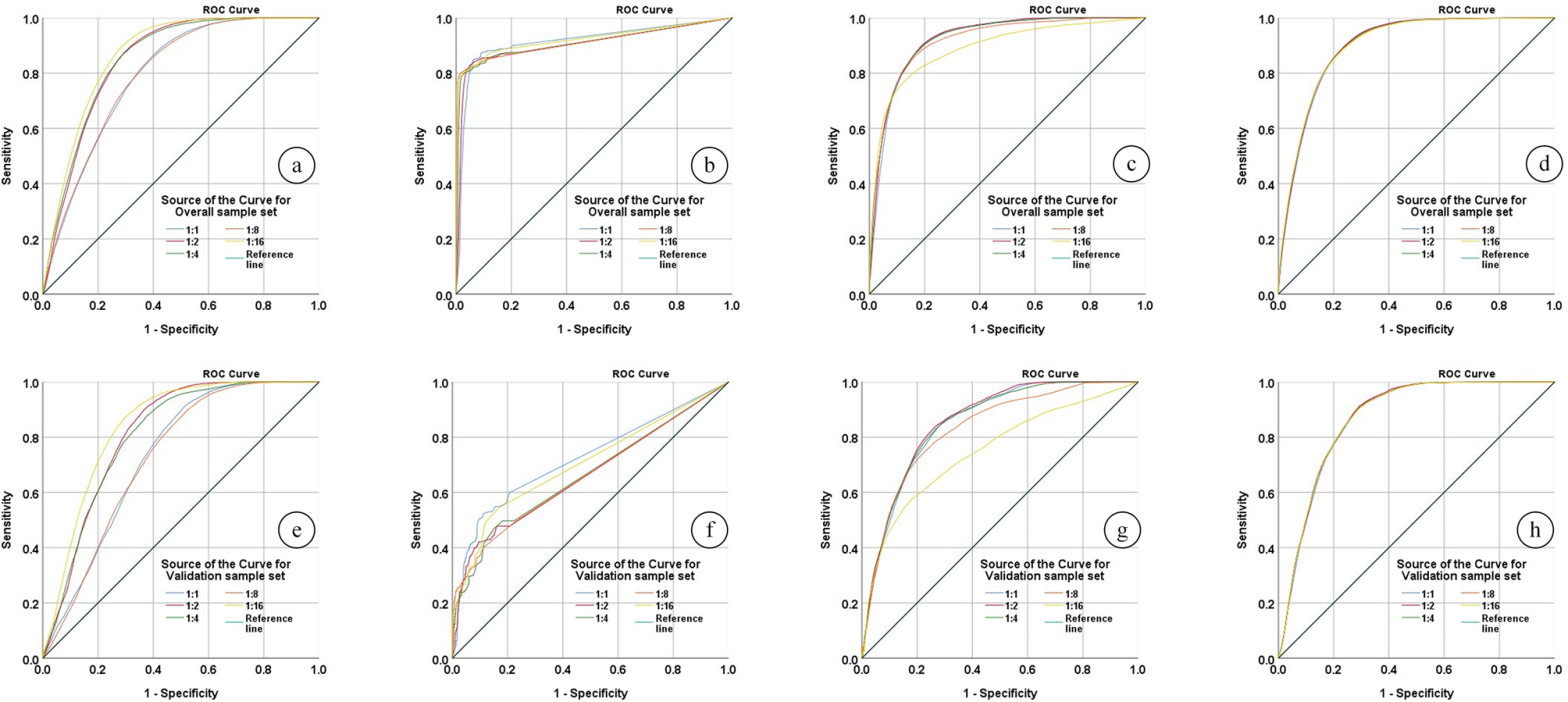
ROC curve analysis for (**a**) Overall sample set by CNN model, (**b**) Validation sample set by CNN model, (**c**) Overall sample set by C5.0 model, (**d**) Validation sample set C5.0 model, (**e**) Overall sample set by SVM model, (**f**) Validation sample set SVM model, (**g**) Overall sample set by LR model, (**h**) Validation sample set LR model.

The ROC curves of LSM results based on five sample sets with different proportions and four types of models are shown in Fig. 11.

The prediction performance of the model in different sample sets is illustrated in Fig. 11. For the CNN model (shown in Fig. 11 (a), (b)), 1:2, 1:4, and 1:16 are closer to the upper left corner than the results of other sample sets; For the C5.0 model (Fig. 11 (c), (d)), the ROC curve in Fig. 11 (c) has a certain range of change. However, in Fig. 11 (d), the ROC curves corresponding to the 1:1 and 1:16 sample sets are closer to the upper left corner than those of the 1:2, 1:4, and 1:8 sample sets, and they are denser and less variable. Compared with other sample sets, the ROC curve of the 1:8 sample set is far from the upper left corner, and the ROC curve of 1:16 is further away from the upper left corner. For the LR model (Fig. 11 (g), (h)), the ROC curves of the two sample sets almost overlap and are all close to the upper left corner. Overall, the sample ratio has a greater impact on the prediction performance of CNN, C5.0, and SVM models, while LR is less affected by changes in the sample ratio.

Table 7 shows the results of the area under the ROC curve for each model trained with an imbalanced sample set to enhance the quantitative analysis.

**Table 7:** The AUC for the four types of models.

Classifiers	Sample set	1:1	1:2	1:4	1:8	1:16
CNN model	Overall	0.793	0.849	0.851	0.794	*0.868*
	Validation	0.732	0.815	0.805	0.726	*0.842*
C5.0 model	Overall	0.914	0.905	0.910	0.913	*0.925*
	Validation	*0.723*	0.662	0.663	0.662	0.704
SVM model	Overall	0.920	*0.924*	0.923	0.917	0.889
	Validation	0.851	*0.857*	0.851	0.830	0.749
LR model	Overall	0.897	*0.899*	*0.899*	0.898	0.898
	Validation	0.867	*0.869*	*0.869*	0.868	0.869

Significant values are in Italic.

The results in the table show that the four types of models achieve qualified prediction performance in any sample set, and the results based on the unbalanced sample set are mostly better than the results on the balanced sample set. The highest AUC values of the CNN, C5.0, SVM, and LR models are 0.868(1:16), 0.842(1:16); 0.925(1:16), 0723(1:1); 0.924(1:2), 0.857(1:2); 0.899(1:2–1:4), 0.869(1:2–1:4), respectively.

#### Five statistical methods

The calculation results of the five statistical methods of overall precision, precision, recall, F-measure, and MCC are shown in Table 8.

**Table 8:** The results of five statistical methods.

Classifiers	Statistical methods	1:1	1:2	1:4	1:8	1:16
CNN model	OA	0.681	0.848	0.912	0.939	*0.940*
	Precision	0.133	0.208	0.254	*0.319*	0.303
	Recall	*0.777*	0.540	0.238	0.016	0
	F-Measure	0.227	*0.300*	0.246	0.031	0.001
	MCC	0.297	*0.347*	0.269	0.079	0.012
C5.0 model	OA	0.932	0.953	0.970	0.979	*0.983*
	Precision	0.465	0.581	0.728	0.855	*0.949*
	Recall	*0.845*	0.808	0.790	0.780	0.757
	F-Measure	0.600	0.676	0.758	0.816	*0.842*
	MCC	0.611	0.674	0.750	0.809	*0.840*
SVM model	OA	0.845	0.892	0.924	0.942	*0.945*
	Precision	0.260	0.329	0.410	0.521	*0.632*
	Recall	*0.857*	0.765	0.608	0.411	0.198
	F-Measure	0.399	0.460	*0.490*	0.459	0.302
	MCC	0.455	0.488	*0.497*	0.464	0.354
LR model	OA	0.799	0.859	0.904	0.931	*0.940*
	Precision	0.212	0.261	0.318	0.393	*0.475*
	Recall	*0.857*	0.730	0.513	0.271	0.097
	F-Measure	0.340	0.385	*0.392*	0.321	0.161
	MCC	0.394	*0.426*	0.410	0.338	0.221

Significant values are in Italic.

The results from the 1:1 sample set to the 1:16 sample set in Table 8 clearly show that the overall precision and accuracy of the four types of models increase, and they all achieve the best results in the 1:16 sample set. The calculation results are as follows: overall accuracy: 0.940, 0.983, 0.945, 0.940; accuracy: 0.303, 0.949, 0.945, 0.475. All models also obtain maximum recall in the 1:1 sample set, where the CNN model obtains 0.777, the C5.0 model obtains 0.845, the SVM model obtains 0.857, and the LR model obtains 0.857. In terms of the changing trend of the recall rate, the calculation results of the four types of models gradually decrease. Only the C5.0 model shows a small decline, and the 1:1 to 1:16 sample set only decreases by 0.088. While the CNN model is in 1.088, the recall in the 1:8 sample set is reduced to only 0.016.

Unlike the above three indicators, except that the F-measure and the MCC of the C5.0 model increase, the calculation results of these two indices of the CNN, SVM, and LR models first increase and then decrease. The CNN model has the best calculation results in the 1:2 sample set, with results of 0.300 and 0.347, respectively; the C5.0 model has the best calculation results in the 1:16 sample set, with 0.842 and 0.840, respectively. The SVM model has the best calculation results in the 1:4 sample set, and the two values are 0.490 and 0.497, respectively. The F-measure of the LR model appears in the 1:4 sample set, which is 0.392, and the MCC is the largest in the 1:2 sample set, which is 0.426. In general, among these five indicators, especially the F-measure and MCC, which can be applied to comprehensively evaluate the classification performance of the model in the presence of an imbalanced sample set^59^, the results of the model on the LSM of the balanced sample set are not the best.

### Specific category precision analysis

**Figure 12:**
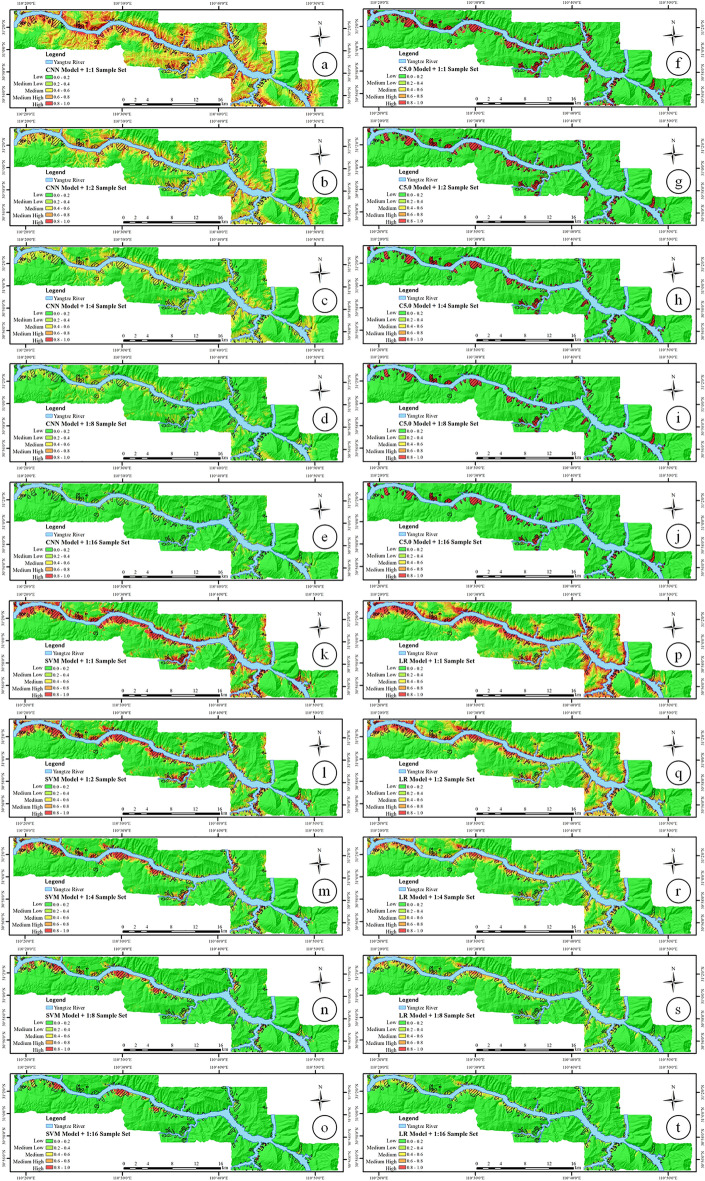
LSZs based on (**a**) 1:1 sample set by CNN model, (**b**) 1:2 sample set by CNN model, (**c**) 1:4 sample set by CNN model, (**d**) 1:8 sample set by CNN model, (**e**) 1:16 sample set by CNN model, (**f**) 1:1 sample set by C5.0 model, (**g**)1:2 sample set by C5.0 model, (**h**) 1:4 sample set by C5.0 model, (**i**) 1:8 sample set by C5.0 model, (**j**) 1:16 sample set by C5.0 model, (**k**) 1:1 sample set by SVM model, (**l**) 1:2 sample set by SVM model, (**m**) 1:4 sample set by SVM model, (**n**) 1:8 sample set by SVM model, (**o**) 1:16 sample set by SVM model, (**p**) 1:1 sample set by LR model, (**q**) 1:2 sample set by LR model, (**r**) 1:4 sample set by LR model, (**s**) 1:8 sample set by LR model, (**t**) 1:16 sample set by LR model (drawn with ArcGIS 10.8 software, and the URL is: https://www.esri.com/en-us/arcgis/about-arcgis/overview).

To increase the readability of the LSI map, all LSIs are divided into five susceptibility categories using the equal interval method according to the calculation results: very low susceptibility (0–0.2), low susceptibility (0.2–0.4), medium susceptibility (0.4–0.6), higher susceptibility (0.6–0.8), and very high susceptibility (0.8–1.0). The SCPA results of LSM based on unbalanced sample sets and different models are shown in Table 9 and Fig. 12.

**Table 9:** Result of SCPA.

Classifiers	Sample set	Category of susceptibility	1:1 (%)	1:2 (%)	1:4 (%)	1:8 (%)	1:16 (%)
CNN model	Overall	Very low	0.24	0.46	1.22	3.60	4.13
		Low	2.56	6.47	12.53	14.61	25.14
		Medium	8.10	16.54	21.77	21.99	31.26
		High	13.48	22.21	25.62	0	0
		Very high	*19.42*	16.55	0	0	0
	Validation	Very low	0.08	0.17	0.51	1.25	1.23
		Low	1.14	2.38	3.72	3.15	5.80
		Medium	2.83	4.76	4.93	2.43	7.97
		High	2.96	4.36	4.62	0	0
		Very high	3.39	*4.06*	0	0	0
C5.0 model	Overall	Very low	1.03	1.24	1.35	1.37	1.51
		Low	9.31	17.50	8.06	18.93	28.25
		Medium	7.98	10.49	17.65	25.71	54.00
		High	11.88	23.43	29.12	49.34	69.56
		Very high	47.81	59.78	76.42	87.12	*95.60*
	Validation	Very low	1.00	1.19	1.28	1.24	1.33
		Low	7.05	14.11	0.95	7.18	7.66
		Medium	1.70	3.33	6.67	0.00	0.00
		High	2.59	9.22	4.54	8.40	22.60
		Very high	9.66	10.39	15.72	31.23	*51.87*
SVM model	Overall	Very low	0.51	0.73	1.27	2.25	4.19
		Low	4.23	7.58	13.14	26.19	47.35
		Medium	7.91	13.31	24.02	31.98	47.00
		High	14.80	24.99	34.43	47.33	49.81
		Very high	37.30	46.22	54.49	64.37	*71.16*
	Validation	Very low	0.40	0.49	0.72	1.04	1.40
		Low	2.18	3.44	4.70	7.06	14.65
		Medium	3.67	5.05	6.90	9.39	11.23
		High	4.74	5.96	8.58	10.30	3.76
		Very high	8.25	*10.54*	10.40	9.07	7.79
LR model	Overall	Very low	0.26	0.55	1.11	1.96	3.33
		Low	3.24	6.34	11.88	20.08	27.59
		Medium	7.79	14.62	22.33	30.01	41.46
		High	16.89	24.53	32.24	44.40	61.34
		Very high	30.74	37.49	48.28	*60.92*	59.65
	Validation	Very low	0.12	0.20	0.42	0.68	1.05
		Low	1.26	2.38	3.99	6.31	8.63
		Medium	2.65	4.84	6.66	8.90	6.05
		High	5.26	7.38	8.70	5.49	2.49
		Very high	*7.60*	7.09	4.75	1.87	0.00

Significant values are in Italic.

According to the results in Table 9, in the CNN model, 4.06% of the results based on the 1:2 sample set with very high susceptibility are higher than 3.39% of the 1:1 sample set, and the results in the 1:4 sample set and later sample sets are all 0. The result of the overall sample set is also 0 in this sample set. The results of the C5.0 model have an obvious increasing trend with the decrease of the sample set, and the best result is in the 1:16 sample set (95.60% of the overall sample set and 51.87% of the validation sample set). The SVM model in the overall sample set also achieves the maximum value of 71.16% in the 1:16 sample set. However, in the validation sample set, the 10.54% result of the 1:2 sample set is the best. In the LR model, the results for the validation sample set range from 7.60% for the balanced sample set to 7.09% for the 1:2 sample set, which is a slight drop in percentage. For the four different models, from SCPA of the very high susceptibility, the results of the imbalanced sample set are better than the results of the traditional training model based on the balanced sample set.

## Discussions

From the ROC curve analysis of the validation sample set and the results of its AUC value, the AUC values of the CNN model, SVM, and LR model increase compared with the results of the 1:1 sample set to the 1:2 sample set, indicating that the three types of models have improved prediction performance in this unbalanced interval. It is worth noting that the AUC results of the C5.0 model in the validation sample set are generally lower, which means that the prediction performance of the C5.0 model in the unbalanced sample set is worse than that of the balanced sample set.

For the calculation results evaluated by the five statistical methods, the OA and the precision of the CNN, the C5.0, the SVM, and the LR models have the best results in the 1:16 sample set. Because all models are affected by the reduction of the sample ratio, the model's ability to distinguish non-landslide samples becomes stronger, and the number of false-positive events is correspondingly reduced, resulting in an increase in the OA and precision of all models. The recall rate reflects the quantitative relationship between TP and FN. The LSM results of the four types of models also have the largest recall rate in the 1:1 sample set. As the landslide and non-landslide sample sets decrease, an increasing amount of landslides are predicted by the model as non-landslides, and the increase in FN events results in a decrease in the recall of all model predictions. F-measure and MCC, as important equilibrium indicators in the evaluation of statistical methods, can effectively measure the performance of the model^59,60^. The results from these two metrics show that the four-class model has better performance than the balanced samples on imbalanced sample sets.

The results of each model in Figs. 10 and 12 show that the four types of models can predict more landslide surfaces using the balanced sample set for LSM compared to the unbalanced sample set. At the same time, because more non-landslide units are predicted as landslide units, the FP number in the prediction results is greater than the number of true positives, and the OA and precision of the four types of models are ultimately lower than the results of other unbalanced sample sets. In addition, judging from the results of the SCPA of the verification sample set, the CNN, the C5.0, and the SVM models that predicted very high susceptibility results in the 1:1 sample set are not the highest. It can be noted that the maximum value of the CNN and SVM models appears in the 1:2 sample set, and it appears in the 1:16 sample set for the C5.0 model. It is worth noting that, in the SCPA, the result of very high LSZ of the CNN model drops from 4.06% in the 1:2 sample set to 0 in the 1:4 sample set. This is because the CNN model quickly calculates the gradient of all parameters by determining the loss function between the real value and the predicted value. This algorithm is used to update the weights, and the gradient generated by the non-landslide samples in the 1:4 sample set is dominant, which increases the shared weights in the fully connected layer that are biased towards predicting non-landslide events, meaning the CNN model begins to bias the prediction of non-landslide events. Although the maximum value of the LR model's very high susceptibility appears in the 1:1 sample set, the LR model's redundant prediction of landslides is significantly reduced. This is illustrated in Fig. 10 (q), where the results of the 1:2 and 1:1 sample set are compared, and the sample set is reduced by only 0.51%. The above phenomenon occurs because the number of non-landslide samples increases within a certain range of unbalanced sample sets so that the number of landslides predicted by models trained on the unbalanced sample set decreases, and the model over-predicts the landslide surface. With a certain degree of correction, the very high susceptibility becomes increasingly concentrated, and the values of the very high susceptibility increase.

**Figure 13:**
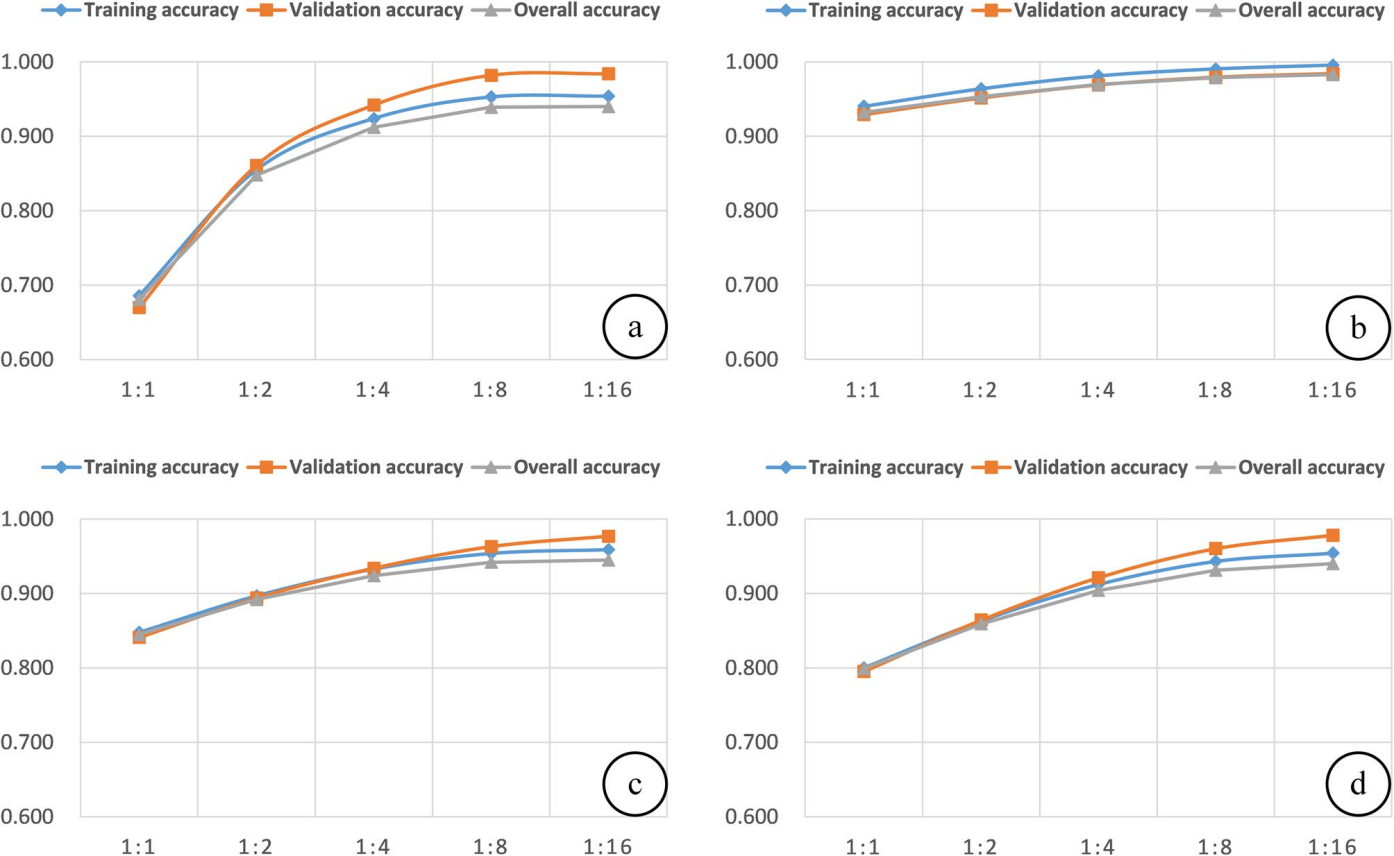
Training accuracy and validation accuracy of (**a**) CNN model, (**b**) C5.0 model, (**c**) SVM model, (**d**) LR model.

To further analyze the impact of the unbalanced sample set on the LSM model and judge its fitting degree, the training accuracy and validation accuracy are added based on the reference OA^22^, as shown in Fig. 13.

According to Fig. 13, the validation accuracy of the CNN, SVM, and LR models is higher than the training accuracy from the 1:2 sample set, indicating that these three types of models have an ideal fitting effect in the 1:2 sample set. The results of the C5.0 model are different from the other three types of models. Its training accuracy is always higher than the validation accuracy, and the values of both are higher, which indicates that the model is overfitting from the 1:1 sample set^61^. In this study, the fitting effect of the C5.0 model in the balanced and unbalanced sample sets is not ideal, so it is impossible to accurately analyze the impact of the unbalanced sample set on its accuracy.

The LSM results of the four types of models were evaluated by three methods: ROC curve and AUC value analysis, five statistical methods, and SCPA. For the C5.0 model, although the sample ratio is smaller, the results of the five statistical methods and the SCPA have better numerical results, and the C5.0 model is fitted in each sample set according to the previous article. The result of analysis shows that the C5.0 model is in a state of overfitting in this study. For the CNN model, SVM model and LR model, to objectively compare the results of the three LSM evaluation methods, a quantitative analysis method–the ranking system is used in Table 10, according to the research method used by Zorlu et al.^62^. This method selects the results of the ROC curve and AUC value analysis, five statistical methods, and SCPA are selected to rank in their categories. For example, in the results of an SVM model, if a sampling proportion has the largest AUC value, it receives a ranking score corresponding to the number of methods it sampled, i.e. 5, the second largest receives 4, and so on.

**Table 10:** Result of ranking system with different sample ratios.

Classifiers	Sample ration	Results				Ranking					Total rank
		Overall		Validation		Overall		Validation		Five statistical methods	
		AUC	SCPA	AUC	SCPA	AUC	SCPA	AUC	SCPA		
CNN model	1:1	0.793	19.42	0.732	3.39	1	5	2	4	2	14
	1:2	0.849	16.55	0.815	4.06	3	4	4	5	5	*21*
	1:4	0.851	0	0.805	0	4	0	3	0	4	11
	1:8	0.794	0	0.726	0	2	0	1	0	3	6
	1:16	0.868	0	0.842	0	5	0	5	0	1	11
SVM model	1:1	0.92	37.3	0.851	8.25	3	1	4	2	3	13
	1:2	0.924	46.22	0.857	10.54	5	2	5	5	4	*21*
	1:4	0.923	54.49	0.851	10.4	4	3	4	4	5	20
	1:8	0.917	64.37	0.83	9.07	2	4	3	3	2	14
	1:16	0.889	71.16	0.749	7.79	1	5	2	1	1	10
LR model	1:1	0.897	30.74	0.867	7.6	3	1	3	5	3	15
	1:2	0.899	37.49	0.869	7.09	5	2	5	4	5	*21*
	1:4	0.899	48.28	0.869	4.75	5	3	5	3	4	20
	1:8	0.898	60.92	0.868	1.87	4	5	4	2	2	17
	1:16	0.898	59.65	0.869	0	4	4	5	1	1	15

Significant values are in Italic.

According to the results in Table 10, CNN model, SVM model and LR model all have the highest total rank in the 1:2 sample set with a score of 21. CNN model has the second highest total rank in the 1:1 sample set, while SVM model and LR model are in the 1:4 sample set. The optimal sample ratio interval can be selected by combining the highest and second highest in total rank, therefore, CNN model has the highest rank in the combination of 1:1 and 1:2 sample sets, and the SVM model and the LR model have the highest rank in the combination of 1:2 and 1:4 sample sets.

The above experimental results show that the results based on the unbalanced sample set are better than the results of the LSM based on the balanced sample set. The experimental results show that using unbalanced sample set for LSM modeling to obtain more accurate prediction results, the LSM results of the CNN model in the sample ratio of 1:1–1:2 and the SVM model and the LR model in the sample ratio of 1:2–1:4 is better than those of the balanced sample.

## Conclusion

LSM using quantitative modeling is closely related to ML. This work analyzed the sample imbalance problem in ML to address unbalanced landslide samples in LSM in depth.

Previous research has demonstrated that models can learn from unbalanced landslide datasets. Zhang et al. found that a model trained with an unbalanced dataset obtained a predictive performance that was comparable to a classifier model trained with a sample-balanced dataset^25^. Researchers have traditionally employed a balanced sample set to train the model in LSM. Although this method could achieve high values in evaluation indicators, such as model precision, recall rate, and AUC value, fundamentally, there are many factors contributing to a good performance, such as using a more advanced model, adjusting and selecting the optimal parameters, or adding more LSM factors. In reality, because the unbalanced sample set in nature is widespread, more suitable for using unbalanced sample set for LSM modeling to obtain more accurate prediction results. The purpose of this study is to show that LSM based on balanced sample sets is one-sided and cannot improve the accuracy of the minority class (i.e., landslide data) by sacrificing the prediction accuracy of the majority class (i.e., non-landslide data). This is useful for LSM models to prevent overfitting and the general overestimation of hazards.

There are two main points worth considering in future research. Firstly, the combination of imbalance and sample size should be considered to explore its effect on LSM; secondly, the relationship between sample proportion and LSM model fit should be studied to determine the sample proportion that can obtain the optimal fit.

## Data Availability

The public data and data processing platform can be downloaded directly through the link provided in Table 1. However, basic geographic data, basic geological data, and landslide distribution data are all confidential data in China. According to the requirements of relevant laws, these confidential data have been decrypted when we use them. Any researchers in related fields that need these decrypted data can contact the corresponding author to obtain them.

## References

[1] Guha-Sapir D, Below R, Hoyois P (2020). EM-DAT: The CRED/OFDA international disaster database. Science.

[2] Peng L, Xu S, Hou J, Peng J (2015). Quantitative risk analysis for landslides: The case of the Three Gorges area, China. Landslides.

[3] Wu X, Ren F, Niu R (2014). Landslide susceptibility assessment using object mapping units, decision tree, and support vector machine models in the Three Gorges of China. Environ. Earth Sci..

[4] Saha AK, Gupta RP, Arora MK (2010). GIS-based Landslide Hazard Zonation in the Bhagirathi (Ganga) Valley, Himalayas. Int. J. Remote Sens..

[5] Aditian A, Kubota T, Shinohara Y (2018). Comparison of GIS-based landslide susceptibility models using frequency ratio, logistic regression, and artificial neural network in a tertiary region of Ambon, Indonesia. Geomorphology.

[6] Akgun A (2012). A comparison of landslide susceptibility maps produced by logistic regression, multi-criteria decision, and likelihood ratio methods: a case study at İzmir, Turkey. Landslides.

[7] Skilodimou HD, Bathrellos GD, Chousianitis K, Youssef AM, Pradhan B (2019). Multi-hazard assessment modeling via multi-criteria analysis and GIS: a case study. Environ. Earth Sci..

[8] Peng L (2014). Landslide susceptibility mapping based on rough set theory and support vector machines: A case of the Three Gorges area, China. Geomorphology.

[9] Mehrabi M, Pradhan B, Moayedi H, Alamri A (2020). Optimizing an adaptive neuro-fuzzy inference system for spatial prediction of landslide susceptibility using four state-of-the-art Metaheuristic techniques. Sensors (Basel).

[10] Chen W, Zhang S, Li R, Shahabi H (2018). Performance evaluation of the GIS-based data mining techniques of best-first decision tree, random forest, and naive Bayes tree for landslide susceptibility modeling. Sci. Total Environ..

[11] Yanbin MA (2022). Machine learning algorithms and techniques for landslide susceptibility investigation: A literature review. J. Civ. Environ. Eng..

[12] Yu, H., Ma, Y., Wang, L., Zhai, Y. & Wang, X. in *2017 IEEE International Conference on Mechatronics and Automation (ICMA).* 40–44.

[13] Lecun Y, Bottou L (1998). Gradient-based learning applied to document recognition. Proc. IEEE.

[14] Wang Y, Fang Z, Hong H (2019). Comparison of convolutional neural networks for landslide susceptibility mapping in Yanshan County, China. Sci. Total Environ..

[15] Li W, Fang Z, Wang Y (2021). Stacking ensemble of deep learning methods for landslide susceptibility mapping in the Three Gorges Reservoirarea, China. Stochastic Environ. Res. Risk Assess..

[16] Fang Z, Wang Y, Peng L, Hong H (2020). Integration of convolutional neural network and conventional machine learning classifiers for landslide susceptibility mapping. Comput. Geosci..

[17] Xiao L, Zhang Y, Peng G (2018). Landslide susceptibility assessment using integrated deep learning algorithm along the China-Nepal highway. Sensors.

[18] Chen Z, Song D, Julie VM, Pourghasemi HR (2021). Landslide susceptibility mapping using statistical bivariate models and their hybrid with normalized spatial-correlated scale index and weighted calibrated landslide potential model. Environ. Earth Sci..

[19] Polykretis C, Chalkias C (2018). Comparison and evaluation of landslide susceptibility maps obtained from weight of evidence, logistic regression, and artificial neural network models. Nat. Hazards J. Int. Soc. Prev. Mitig. Nat. Hazards.

[20] Song Y (2018). Landslide susceptibility mapping based on weighted gradient boosting decision tree in Wanzhou section of the three gorges reservoir area (China). Int. J. Geo-Inform..

[21] Ying W, Lin Q, Shi P (2018). Spatial pattern and influencing factors of landslide casualty events. J. Geog. Sci..

[22] Gao H, Fam PS, Tay LT, Low HC (2020). Comparative landslide spatial research based on various sample sizes and ratios in Penang Island, Malaysia. Bull. Eng. Geol. Environ..

[23] Zhi WM, Guo HP, Fan M (2013). Sample size on the impact of imbalance learning. Adv. Mater. Res..

[24] Wang Y (2019). Optimizing the predictive ability of machine learning methods for landslide susceptibility mapping using SMOTE for Lishui City in Zhejiang Province, China. Int. J. Environ. Res. Public Health.

[25] Zhang H (2022). Combining a class-weighted algorithm and machine learning models in landslide susceptibility mapping: A case study of Wanzhou section of the Three Gorges Reservoir, China. Comput. Geosci..

[26] Aktaş H, San B (2019). Landslide susceptibility mapping using an automatic sampling algorithm based on two level random sampling. Comput. Geosci..

[27] Chang Z (2020). Landslide susceptibility prediction based on remote sensing images and GIS: Comparisons of supervised and unsupervised machine learning models. Remote Sens..

[28] Survey HPG (1997). Cartographer Geological Map of Zigui and Badong COUNTY (1:50,000).

[29] Reservoir HoPaCoG-HiAoTG, cartographer 1:10,000 geological hazard mapping database2011.

[30] Yu X, Gao H (2020). A landslide susceptibility map based on spatial scale segmentation: A case study at Zigui-Badong in the Three Gorges Reservoir Area, China. PLOS ONE.

[31] Bai S-B (2010). GIS-based logistic regression for landslide susceptibility mapping of the Zhongxian segment in the Three Gorges area, China. Geomorphology.

[32] Chen J, Zeng Z, Jiang P, Tang H (2015). Deformation prediction of landslide based on functional network. Neurocomputing.

[33] Pham BT, Shirzadi A, Tien BD, Prakash I, Dholakia MB (2018). A hybrid machine learning ensemble approach based on a Radial Basis Function neural network and Rotation Forest for landslide susceptibility modeling: A case study in the Himalayan area, India. Int. J. Sedim. Res..

[34] Yu, X. *Study on the Landslide Susceptibility Evaluation Method Based on Multi-source Data and Multi-scale Analysis Doctor thesis (China University of Geosciences, 2016).* (2016).

[35] Hong H, Liu J, Zhu AX (2020). Modeling landslide susceptibility using LogitBoost alternating decision trees and forest by penalizing attributes with the bagging ensemble. Sci. Total Environ..

[36] Yu X, Zhang K, Song Y, Jiang W, Zhou J (2021). Study on landslide susceptibility mapping based on rock-soil characteristic factors. Sci. Rep..

[37] Dormann, C. G., Elith, J., Bacher, S. & Lautenback, S. Collinearity: a review of methods to deal with it and a simulation study evaluating their performance. (2012).

[38] Gao H, Fam PS, Tay LT, Low HC (2020). Three oversampling methods applied in a comparative landslide spatial research in Penang Island, Malaysia. SN Appl. Sci..

[39] Kira, K. & Rendell, L. A. in *Tenth National Conference on Artificial Intelligence.*

[40] Fang Z, Wang Y, Peng L, Hong H (2020). A comparative study of heterogeneous ensemble-learning techniques for landslide susceptibility mapping. Int. J. Geogr. Inform. Sci..

[41] Vapnik, V. N. *The Nature of Statistical Learning Theory*. (The nature of statistical learning theory, 1995).

[42] Chong X, Dai F, Xu X, Yuan HL (2012). GIS-based support vector machine modeling of earthquake-triggered landslide susceptibility in the Jianjiang River watershed, China. Geomorphology.

[43] Tang RX, Yan EC, Wen T, Yin XM, Tang W (2021). Comparison of logistic regression, information value, and comprehensive evaluating model for landslide susceptibility mapping. Sustainability.

[44] Haibo H, Garcia EA (2009). Learning from Imbalanced Data. IEEE Trans. Knowl. Data Eng..

[45] Pourghasemi HR, Rahmati O (2018). Prediction of the landslide susceptibility: Which algorithm, which precision?. CATENA.

[46] Sameen MI, Pradhan B, Lee S (2020). Application of convolutional neural networks featuring Bayesian optimization for landslide susceptibility assessment. Catena.

[47] Nath RR, Sharma ML, Goswami A, Sweta K, Pareek N (2021). Landslide susceptibility zonation with special emphasis on tectonic features for occurrence of landslides in lower Indian Himalaya. Science.

[48] Demir G, Aytekin M, Akgün A, İkizler SB, Tatar O (2012). A comparison of landslide susceptibility mapping of the eastern part of the North Anatolian Fault Zone (Turkey) by likelihood-frequency ratio and analytic hierarchy process methods. Nat. Hazards.

[49] Nath RR, Das N, Satyam DN (2021). Impact of main boundary thrust (MBT) on landslide susceptibility in Garhwal Himalaya: A case study. Indian Geotech. J..

[50] Polykretis C, Ferentinou M, Chalkias C (2015). A comparative study of landslide susceptibility mapping using landslide susceptibility index and artificial neural networks in the Krios River and Krathis River catchments (northern Peloponnesus, Greece). Bull. Eng. Geol. Environ..

[51] Ciurleo M, Cascini L, Calvello M (2017). A comparison of statistical and deterministic methods for shallow landslide susceptibility zoning in clayey soils. Eng. Geol..

[52] Sadr MP, Maghsoudi A, Saljoughi BS (2014). Landslide susceptibility mapping of Komroud Sub-basin using fuzzy logic approach. Geodynamics.

[53] Hong (2018). Landslide susceptibility mapping using J48 Decision Tree with AdaBoost, Bagging and Rotation Forest ensembles in the Guangchang area (China). Catena Interdis. J. Soil Sci. Hydrol..

[54] Fang Z, Wang Y, Peng L, Hong H (2020). Integration of convolutional neural network and conventional machine learning classifiers for landslide susceptibility mapping. Comput. Geosci..

[55] Jebur MN, Pradhan B, Tehrany MS (2014). Optimization of landslide conditioning factors using very high-resolution airborne laser scanning (LiDAR) data at catchment scale. Remote Sens. Environ..

[56] Aas A, Js B, Fj B, Sl C (2021). Landslide susceptibility hazard map in southwest Sweden using artificial neural network. CATENA.

[57] Pourghasemi HR, Pradhan B, Gokceoglu C (2012). Application of fuzzy logic and analytical hierarchy process (AHP) to landslide susceptibility mapping at Haraz watershed, Iran. Nat. Hazards.

[58] Pourghasemi HR, Rossi M (2017). Landslide susceptibility modeling in a landslide prone area in Mazandarn Province, north of Iran: a comparison between GLM, GAM, MARS, and M-AHP methods. Theoret. Appl. Climatol..

[59] Sabri B, Fethi J, Mohammed EA, Quan Z (2017). Optimal classifier for imbalanced data using Matthews correlation coefficient metric. Plos One.

[60] Liu XY, Wu J, Zhou ZH (2009). Exploratory undersampling for class-imbalance learning. IEEE Trans. Syst. Man Cybern..

[61] Wei XS, Wang P, Liu L, Shen C, Wu J (2019). Piecewise classifier mappings: Learning fine-grained learners for novel categories with few examples. IEEE Trans. Image Process..

[62] Zorlu K, Gokceoglu C, Ocakoglu F, Nefeslioglu HA, Acikalin S (2008). Prediction of uniaxial compressive strength of sandstones using petrography-based models. Eng. Geol..

